# Supplementary material for: Feasibility of Encord Artificial Intelligence Annotation of Arterial Duplex Ultrasound Images
Source: Diagnostics (Basel). 2023 Dec 25;14(1):46. doi: 10.3390/diagnostics14010046 (PMC10795888; doi:10.3390/diagnostics14010046)

Supplemental Figure S1. Example Screenshot of Encord platform training log for the 80 image model. The upper left hand corner is labeled the model with segmentation, PyTorch, and Mask Region Based Convolutional Neural Networks (MASK_RCNN). The graph displayed shows model loss on the y axis and Epoch on the y axis.

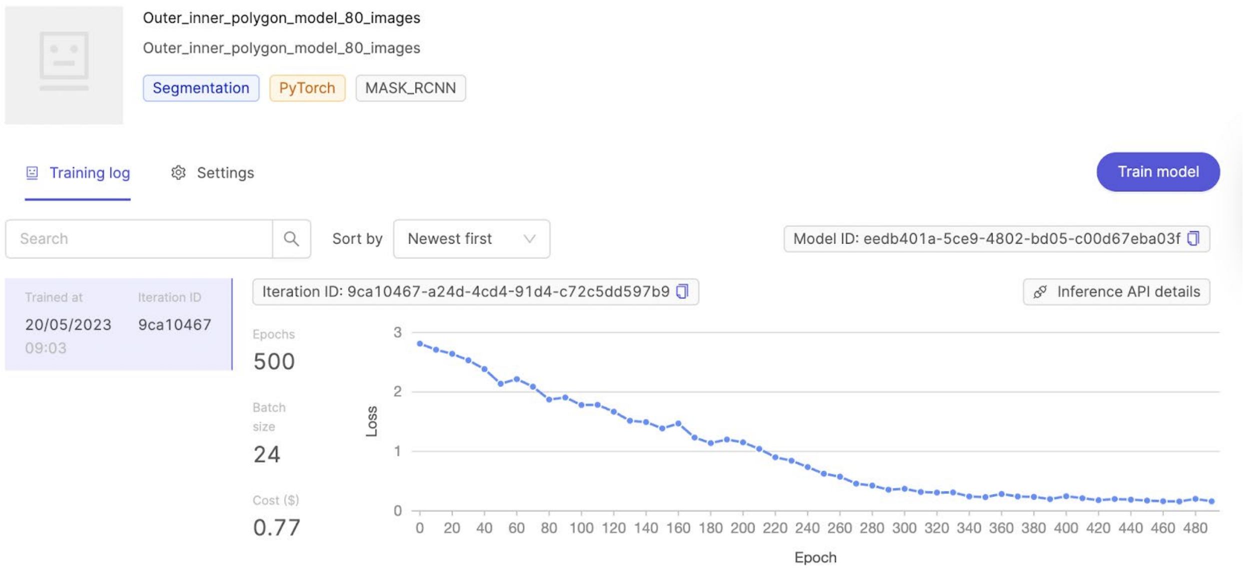

Supplement: Supplementary file 1 [file diagnostics-14-00046-s001.zip › diagnostics-2716507-supplementary.pdf]
